# 58210 Perspectives and Guidance for Mobile Health Self-Management Intervention Developers from Adolescents and Young Adults with Chronic Illnesses: A Qualitative Study

**DOI:** 10.1017/cts.2021.535

**Published:** 2021-03-30

**Authors:** Caitlin S. Sayegh, Ellen Iverson, Clarissa Newman, Diane Tanaka, Ellen F. Olshansky, Clarisa Wijaya, Marvin Belzer

**Affiliations:** 1 University of Southern California Keck School of Medicine; 2 University of Southern California; 3 University of Southern California Suzanne Dworak-Peck School of Social Work Department of Nursing and Sue & Bill Gross School of Nursing at UCI

## Abstract

ABSTRACT IMPACT: The perspectives and guidance from adolescents and young adults (AYA) reported in this study could inform the evidence-based development and delivery of mobile health (mHealth) interventions to improve the health of AYA with chronic diseases. OBJECTIVES/GOALS: To elicit advice from AYA with chronic healthcare needs regarding if and how mHealth interventions could effectively promote illness self-management skills. We selected this goal because including the user perspective from the beginning of the design process could lead to greater future adoption. METHODS/STUDY POPULATION: We purposively recruited AYA patients from a pediatric hospital with heterogeneous chronic illnesses to identify universal chronic disease views rather than condition-specific perspectives. We conducted qualitative face-to-face semi-structured interviews with (N = 19) AYA between 16 and 20 years old (63.2% Latinx; 21.1% Black; 10.5% White; 5.3% Multiracial). Using ATLAS.ti, three coders completed thematic analysis to inform a conceptual framework on how AYA believe mHealth interventions could promote the development and maintenance of self-management skills. Member checking was conducted over the phone to obtain participant feedback on themes to enhance the validity of qualitative results. RESULTS/ANTICIPATED RESULTS: Results suggest that AYA develop self-management skills through several strategies, including 1) getting organized, 2) ‘making it work for me’ and 3) keeping the ‘right’ mentality. AYA described developing these strategies through: 1) receiving social support, 2) accessing helpful tools and technologies, and 3) going through a maturation process. They provided recommendations for how mHealth interventions could improve this process, including: 1) ‘what’ recommendations, describing the content or active ingredients that should be included in mHealth interventions, and 2) ‘how’ recommendations, describing the technological aspects or style in which the interventions should be delivered. DISCUSSION/SIGNIFICANCE OF FINDINGS: The results suggest that an appealing mHealth intervention could increase the support for AYA patients to proactively acquire self-management skills, avoiding trial-and-error or uneven access to guidance. Improving self-management could prevent poor health outcomes and increase quality of life.

